# A case of Marfanoid-progeroid-lipodystrophy syndrome: experimental proof of skipping exons and escaping nonsense-mediated decay

**DOI:** 10.1038/s41439-023-00255-8

**Published:** 2023-10-16

**Authors:** Takahito Moriwaki, Mitsuo Masuno, Miho Nagata, Yasuki Ishihara, Yohei Miyashita, Yoshihiro Asano, Kayo Takao, Kazumi Tawa, Yasuko Yamanouchi, Atsushi Miki, Takanobu Otomo

**Affiliations:** 1 Department of Molecular and Genetic Medicine, Kawasaki Medical School, Kurashiki, Japan; 2 Department of Medical Genetics, Kawasaki Medical School Hospital, Kurashiki, Japan; 3 Genetic Counseling Program, Graduate School of Health and Welfare, Kawasaki University of Medical Welfare, Kurashiki, Japan; 4 Department of Cardiovascular Medicine (IRUD Analysis Center), Osaka University Graduate School of Medicine, Suita, Japan; 5 Department of Ophthalmology, Kawasaki Medical School, Kurashiki, Japan

**Keywords:** Clinical genetics, Diseases, Genetics research

## Abstract

We report a Japanese patient with tall stature, dolichocephaly, prominent forehead, narrow nasal ridge, mild retrognathia, subcutaneous fat reduction, bilateral entropion of both eyelids, high arched palate, long fingers, and mild hyperextensible finger joints as a case of Marfanoid-progeroid-lipodystrophy syndrome. Genetic investigation revealed a heterozygous variant NC_000015.10(NM_000138.5):c.8226+5G>A in the *FBN1* gene. Skipping of exon 65 and escaping nonsense-mediated decay followed by frameshift were experimentally confirmed in the proband’s mRNA.

Marfanoid-progeroid-lipodystrophy syndrome (MFLS, MIM #616914)^1^ is caused by heterozygous variants affecting exon 65 near the 3’ terminus of the *FBN1* gene, which is known as one of the causative genes for Marfan syndrome. MFLS is characterized by clinical features including fetal growth retardation, birth before 40 weeks, generalized lack of subcutaneous fat, hyperextensible joints, long fingers, and severe myopia^2,3^. The phenotypic association between MFLS and Marfan syndrome^4^ is variable, and MFLS’s appearance of premature aging is not due to actual premature aging but rather due to low subcutaneous fat. The C-terminal cleavage product of profibrillin (encoded by *FBN1*), which is named asprosin, acts as a glucogenic protein hormone^5^. Asprosin is a centrally acting orexigenic hormone, as confirmed in patients and a mouse model^6^, and is of interest as a potential therapeutic target molecule for obesity and diabetes. In patients with frameshift variants near the 3’ end of *FBN1*, if the mutant transcripts escape nonsense-mediated decay (NMD), the amount of truncated profibrillin may be relatively maintained, although asprosin is lost. Reduction of asprosin lowers blood glucose and insulin and is assumed to contribute to the impaired metabolic state leading to lipodystrophy. The effect of the genomic variant on the transcribed mRNA is still unknown. The purpose of this study was to determine the molecular mechanisms by which mutant transcripts define the phenotype of this disease.

The proband was a 9-year-7-month-old Japanese girl. She was the fourth child of a 33-year-old mother and a nonconsanguineous 35-year-old father. One pregnancy of the mother ended in a spontaneous abortion. Her parents, elder brother, and twin sisters were in good health. Her mother and father were 156.0 cm and 169.5 cm tall, respectively, which are within the normal reference range in Japan. Fetal growth stopped around 32 weeks, suggesting placental insufficiency. The proband was born at 35 weeks and 2 days’ gestation by (repeat) cesarean section with an Apgar score of 8 at 1 minute. Her birth weight was 1,556 g (−2.6 SD), height was 45.4 cm (0.0 SD), and head circumference was 33.2 cm (+1.0 SD). No abnormality was reported in the neonatal screening of inherited metabolic diseases and auditory brainstem response. During the early neonatal period, bilateral entropion of the upper and lower lids and dolichocephaly were noted. Because she had severe corneal epithelial damage, she underwent surgery to correct bilateral entropion at Kawasaki Medical School Hospital at the age of 1 year 4 months. She walked without help at 18 months of age, and her psychomotor development was within the normal range. Mild mitral regurgitation was identified at 2 years of age. She was followed up in another hospital by echocardiography, showing stable mild mitral regurgitation without dilation of the aortic root. She was consulted by the Department of Medical Genetics of Kawasaki Medical School Hospital at 5 years 9 months of age for the first time for an undiagnosed condition and a poor appetite. The main clinical manifestations included dolichocephaly, prominent forehead, narrow nasal ridge, mild retrognathia, subcutaneous fat reduction, bilateral entropion (postoperative), high arched palate, long fingers, positive wrist and thumb signs, plain pes planus, and mild hyperextensible finger joints (Fig. 1). Ophthalmological evaluations revealed myopic astigmatism (Rt:−2.5 diopter sphere with −2.5 diopter cylinder at 90 degrees; Lt: −3.0 diopter sphere with −2.0 diopter at 70 degrees), without ectopia lentis. At 9 years 7 months of age, she was 151.6 cm (+2.8 SD) in height, weighed 29.0 kg (−0.3 SD), and had a head circumference of 55.3 cm (+2.2 SD). Her arm span was 158.0 cm, body mass index (BMI) was 12.6 kg/m^2^, and body fat percentage was 9.4%. The systemic score of Marfan syndrome^7^ was calculated as 5. Her karyotype using G-banding was 46,XX. Chromosomal microarray analysis (GenetiSure Dx Postnatal Assay, Agilent) showed no pathogenic variants. Subsequently, whole-exome sequencing was performed as part of the research project “Initiative on Rare and Undiagnosed Diseases; IRUD^8^,” using a trio of samples from the proband and parents. A heterozygous variant, NC_000015.10(NM_000138.5):c.8226+5G>A, was identified in intron 65 of the *FBN1* gene of the proband (Fig. 2a), suggesting that it was a de novo occurrence in the proband. This genomic variant was confirmed using Sanger sequencing (Fig. 2b).

**Fig. 1:**
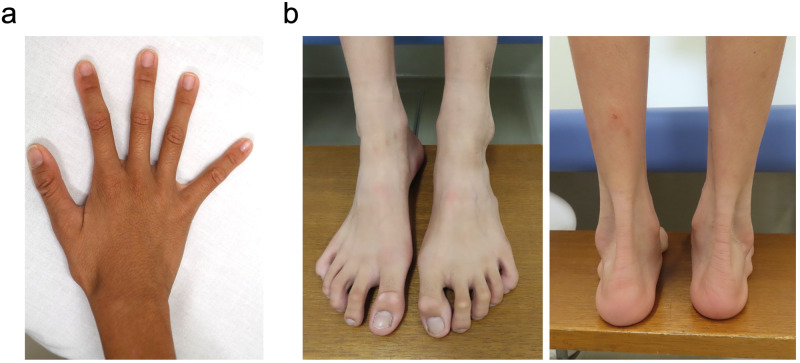
Appearances of the fingers and legs of the proband. **a**, **b** Long fingers of the hands and foot, without hindfoot deformity. The use of photographs was approved by the proband and her parents in writing.

**Fig. 2:**
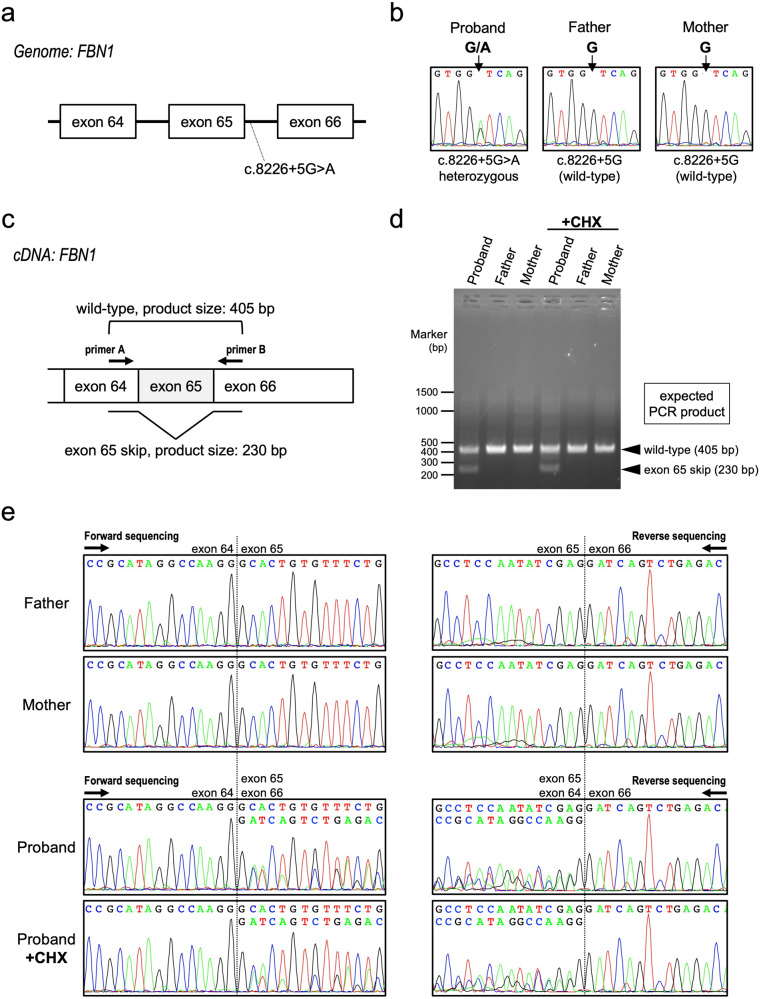
Molecular analyses of FBN1. **a** Scheme of the genomic variant located in intron 65. **b** Sanger sequencing of genomic DNA. The heterozygous c.8226+5G>A variant was identified in the proband. **c** Scheme of PCR in cDNA. PCR primers A and B are indicated by arrows. The expected sizes of the amplicon are 405bp in wild-type and 230 bp in exon 65-skipped mRNA. **d** Gel electrophoresis of PCR products. PCR was performed on cDNA synthesized from the mRNA of cycloheximide (CHX)-treated and untreated lymphoblastoid cell lines. Extra small bands were observed in the proband, which is consistent with the size skipped by exon 65, as well as large bands common to parents that are considered consistent with the wild-type. **e** Sequencing analyses of cDNA. cDNA sequences were read in both directions using primers A (forward sequencing) and B (reverse sequencing) with the PCR products. In the proband, two overlapping chromatograms were observed from the junctions of exons 64/65 and 65/66, indicating the skipping of exon 65. The exon-65-skipped mRNA did not seem to be degraded by NMD because CHX treatment did not change the wave heights of the double chromatograms.

To investigate the effect of the genomic variant on the transcribed mRNA, lymphoblastoid cell lines treated with cycloheximide (CHX) were used. CHX is known to inhibit NMD, and we performed this experiment with an established method^9^. We amplified a fragment containing exon 64 – exon 65 – exon 66 from cDNA using primers A and B (Fig. 2c) and found that the sample from the proband exhibited a small PCR product in addition to the expected full-size product (Fig. 2d). Judging from its size, this small band was assumed to be the 230 bp product skipped by exon 65. The amount of the small band was not changed by CHX treatment. Sanger sequencing revealed that the mRNA from the proband contained both the wild-type and exon 65-skipped sequence, which was confirmed by our detection of both boundaries between exon 64/65 and exon 65/66 (Fig. 2e). If NMD occurs on the mutant allele, a change in the amount of mutant RNA is observed via treatment with CHX. However, in our case, CHX treatment did not alter the patterns of the chromatogram in the proband, which suggests that the mutant RNA was not the target of NMD (Fig. 2e, lowest panels). Given all this, we conclude that the c.8226+5G>A variant in the *FBN1* gene led to the skipping of exon 65, causing a frameshift, and that this mutant transcript escaped NMD.

To our knowledge, eight cases of MFLS associated with heterozygous variants near the 3’ terminus of the *FBN1* gene have been reported^10–16^. Among them, five cases had exonic insertion or deletion variants causing a frameshift in exon 65 or 66 of *FBN1*. The other three cases had intronic single-nucleotide substitutions: one case with NC_000015.10(NM_000138.5):c.8226+1G>A and two cases with NC_000015.10(NM_000138.5):c.8226+1G>T, both of which were located in intron 65 of the *FBN1* gene. Skipping of exon 65 in mRNA (cDNA) due to an intronic variant has been experimentally confirmed in one case of c.8226+1G>A^14^. It is thought that all of these MFLS variants lead to a frameshift generating premature termination codons. Since these variants are located in the last 50 nt of the final exon boundary, the mRNAs may escape NMD. NMD escape has been predicted by the in silico tool NMDescPredictor for the five exonic variants but has not been performed for intronic variants^16^. There is no experimental proof of NMD escape for any reported variant before the current one.

Apart from these MFLS reports, the c.8226+5G>A variant, the same as our patient had, has been reported in two earlier cases. One was found by genetic screening of patients with craniosynostosis using whole-genome sequencing^17^. The patient showed exorbitism, ligamentous laxity, recurrent inguinal hernia, tall stature, lens subluxation, and mild aortic dilatation at 8 years old, as well as sagittal and metopic suture synostosis, which overlap with the phenotypes of Marfan syndrome. The other was a case presented in a genetic congress of cardiovascular diseases^18^. The patient showed a failure to thrive, prominent forehead, malar hypoplasia, pectus carinatum, arachnodactyly, hypermobility, delayed motor development, frequent pulmonary infections, a striking progeroid appearance, and normal aortic root diameter. The case was discussed as a progeroid variant of Marfan syndrome. The phenotypic differences between our case and these two previous cases are interesting, but we believe that all three cases are within the range of *FBN1*-related diseases.

To summarize our molecular investigation, this is the first experimental confirmation of exon 65 skipping due to the c.8226+5G>A variant in the *FBN1* gene associated with MFLS. NMD escape was also confirmed experimentally for the first time in a clinical samples of a MFLS patient.

MFLS is an extremely rare medical condition caused by heterozygous pathogenic variants in the *FBN1* gene, with characteristic clinical manifestations of severe partial lipodystrophy, premature birth with accelerated linear growth disproportionate to weight gain, and a progeroid appearance with distinct facial features, as well as a variety of clinical manifestations partially overlapping with Marfan syndrome^1–4^. The clinical features of our case are consistent with previous MFLS descriptions. Craniosynostosis is rare in *FBN1*-related Marfan syndrome but is reported in several cases in MFLS^13,17^. As we described, a patient with the same genetic variant as ours was diagnosed via genetic screening of craniosynostosis^17^. Although craniosynostosis was not confirmed in our case because no CT was taken, the presence of dolichocephaly is collateral evidence. On the other hand, the eyelid entropion seen in the present case seems to be a rare manifestation of MFLS. Only one case of eyelid entropion has previously been reported^14^. In that case, skipping of exon 65 was experimentally confirmed in the mRNA, although the genetic mutation was different. Since the clinical symptoms were consistent with previous cases and the genetic mutation was confirmed, we believe that this case can be definitively diagnosed as a case of MFLS.

## Data Availability

The relevant data from this Data Report are hosted at the Human Genome Variation Database at 10.6084/m9.figshare.hgv.3333.
